# Supplementary material for: Yiai Fuzheng decoction inhibits triple-negative breast cancer by remodeling the immune microenvironment
Source: Front Immunol. 2025 Sep 30;16:1615631. doi: 10.3389/fimmu.2025.1615631 (PMC12518410; doi:10.3389/fimmu.2025.1615631)
Supplement: Supplementary file 2 [file DataSheet1.zip › Supplementary File 2/Treatment/C_vs_D_vs_H/01_Basic_Statistics/Metabolite_Class_Statistics/Class_Barplot_by_Group.pdf]

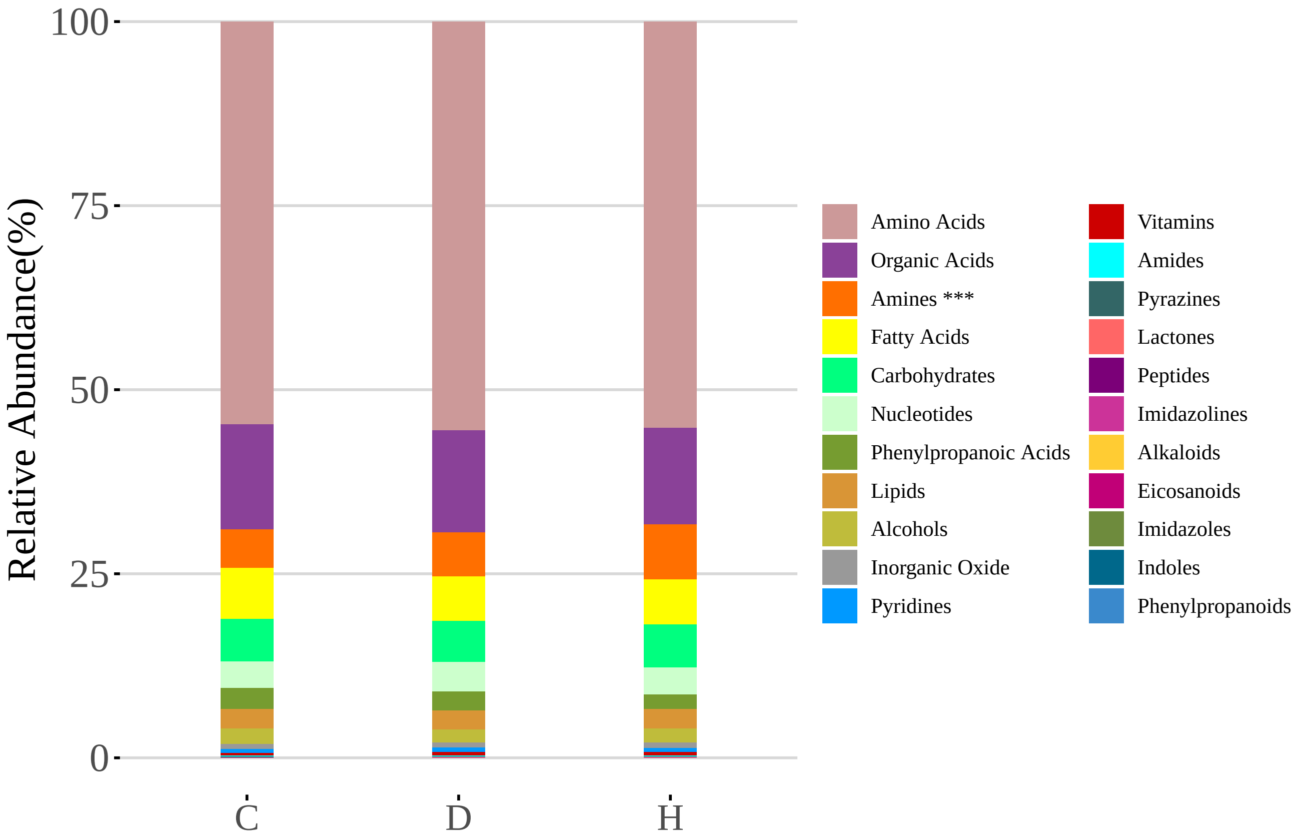

Relative Abundance(%)
100
75
50
25
0
C
D
H
Amino Acids
Organic Acids
Amines ***
Fatty Acids
Carbohydrates
Nucleotides
Phenylpropanoic Acids
Lipids
Alcohols
Inorganic Oxide
Pyridines
Vitamins
Amides
Pyrazines
Lactones
Peptides
Imidazolines
Alkaloids
Eicosanoids
Imidazoles
Indoles
Phenylpropanoids
